# Automated Segmentation of Median Nerve in Dynamic Sonography Using Deep Learning: Evaluation of Model Performance

**DOI:** 10.3390/diagnostics11101893

**Published:** 2021-10-14

**Authors:** Chueh-Hung Wu, Wei-Ting Syu, Meng-Ting Lin, Cheng-Liang Yeh, Mathieu Boudier-Revéret, Ming-Yen Hsiao, Po-Ling Kuo

**Affiliations:** 1 Department of Physical Medicine and Rehabilitation, National Taiwan University Hospital Hsin-Chu Branch, Hsinchu 300, Taiwan; b88401062@ntu.edu.tw (C.-H.W.); b96401093@gmail.com (M.-T.L.); 2 Department of Physical Medicine and Rehabilitation, National Taiwan University Hospital, College of Medicine, National Taiwan University, Taipei 100, Taiwan; myferrant@gmail.com; 3 Graduate Institute of Biomedical Electronics and Bioinformatics, National Taiwan University, Taipei 106, Taiwan; r06945032@ntu.edu.tw (W.-T.S.); r08945006@ntu.edu.tw (C.-L.Y.); 4 Centre Hospitalier de l’Université de Montréal, Montreal, QC H2X 3E4, Canada; mathieu.boudier-reveret@umontreal.ca; 5 Department of Electrical Engineering, National Taiwan University, Taipei 106, Taiwan

**Keywords:** carpal tunnel syndrome, nerve segmentation, ultrasound, deep learning

## Abstract

There is an emerging trend to employ dynamic sonography in the diagnosis of entrapment neuropathy, which exhibits aberrant spatiotemporal characteristics of the entrapped nerve when adjacent tissues move. However, the manual tracking of the entrapped nerve in consecutive images demands tons of human labors and impedes its popularity clinically. Here we evaluated the performance of automated median nerve segmentation in dynamic sonography using a variety of deep learning models pretrained with ImageNet, including DeepLabV3+, U-Net, FPN, and Mask-R-CNN. Dynamic ultrasound images of the median nerve at across wrist level were acquired from 52 subjects diagnosed as carpal tunnel syndrome when they moved their fingers. The videos of 16 subjects exhibiting diverse appearance and that of the remaining 36 subjects were used for model test and training, respectively. The centroid, circularity, perimeter, and cross section area of the median nerve in individual frame were automatically determined from the inferred nerve. The model performance was evaluated by the score of intersection over union (IoU) between the annotated and model-predicted data. We found that both DeepLabV3+ and Mask R-CNN predicted median nerve the best with averaged IOU scores close to 0.83, which indicates the feasibility of automated median nerve segmentation in dynamic sonography using deep learning.

## 1. Introduction

Entrapment neuropathy, the spatial constraint of a peripheral nerve by its surrounding tissues, is usually debilitating by bringing about numbness and weakness of the innervated tissues. Carpal tunnel syndrome (CTS) is the most common entrapment neuropathy and involves the entrapment of median nerve (MN) at across wrist level [1,2]. Traditionally, the confirmatory diagnosis and severity evaluation of CTS are conducted using nerve conduction study (NCS) and needle electromyography. However, they are invasive and may be unacceptable by the patients. Even in symptomatic CTS patients, normal results in NCSs are not uncommon [3]. A non-invasive and easily accessible diagnostic tool is needed.

Ultrasonography (US) has been proposed as a promising complement for the diagnosis and evaluation of CTS in conjunction with NCS [4,5], particularly in conditions with normal NCS results [6,7,8]. Morphological changes, such as enlargement of cross-sectional area (CSA) of the MN at the pisiform level measured with US [9], might indicate CTS with the same accuracy as electrodiagnostic studies [10]. In addition, an increasing line of evidence suggests that dynamic US may reveal abnormal motion patterns of the entrapped MN [11,12,13,14]. Compared to healthy subjects, patients with CTS exhibited significantly less deformation in terms of circularity and less displacement magnitude of the MN during finger and wrist motions [11,12,13]. Furthermore, the degrees of changes in either MN morphology or displacement during wrist motion may be related to CTS severity [14,15]. However, currently the tracking of MN in consecutive US images still depends on manual recognition and delineation, which requires massive human labors and makes it hard to implement the analysis clinically. Moreover, images acquired in dynamic US as the nerve is moved is usually noisier than static images because of the motion artifacts [16], which further increases the difficulty in manual tracking. An appealing solution is to employ machine learning to automatically segment the tracked nerve in the images.

In musculoskeletal US, deep-learning (DL) has been emerging as the leading machine-learning tool to segment a variety of anatomical structures, such as muscles [17,18], nerves [16,19,20,21,22,23,24,25], and spinous processes [26]. Because of their small size and speckle noises, differentiation of nerves from adjacent tissues is generally a challenging task in clinical practice [21], which drives the development of DL-based approaches to assist the nerve visualization. The reported applications include automatic segmentation of the brachial plexus at the axillary level [21,23,24,25], and the femoral nerve at the level of the femoral crease [22], to assist training of nerve block. Most of the approaches employed models based on convolutional neural network (CNN) [19], particularly U-Net [16,20,21,22,23,24,25], an architecture of symmetric u-shape to overcome the progressive loss of feature resolution when the structure increases depth, by combining the spatial information of extracted features at low level with the high-level semantic information [27]. However, there was few research evaluating nerve segmentation using later DL models, such as DeepLabv3+, which improves the preservation of the spatial information during the extraction of the semantic features [28]. Moreover, there was scanty report focusing on nerve segmentation in dynamic US, which is expected to be more challenging than static US because of the motion artifacts. Hafiane et al. utilized CNN reinforced by comparing the spatial and temporal consistency of US acquired by probe movement to automatically segment MN at forearm level [19]. However, the nerve was indeed segmented using an active contouring model rather than DL-based approaches. In addition, the procedure that collects the image sequence by probe movement cannot be classified as dynamic US, which is acquired when the subject performs a particular movement as the probe is held stationary; the quality of image acquired by the former is expected to be less noisy because the change in the image content is fully controlled by the examiner. Furthermore, application employing state-of-the-art DL models to automatically extract the morphological dynamic of MN during fingers motion in patients with CTS is barely addressed. Festen et al. recently reported the automatic segmentation of MN in dynamic US using U-Net [16]. The data were collected from 99 CTS patients and better results were found in images acquired during finger flexion. However, the morphological dynamic of MN during fingers motion was not addressed. A U-Net based model combined with convolutional long short-term memory to segment MN and extract the morphological data was recently reported, but there were only two patients enrolled [20]. Besides, a main obstacle hindering the blooming of DL systems in musculoskeletal US applications is the scant access to well-labeled US datasets for model pretraining, mostly resulting from the expensive annotation by human experts [29,30]. A popular approach addressing this challenge is to transfer knowledge learned from publicly available, large datasets of natural images, such as ImageNet, to the US domain. This process is generally accomplished by using models pretrained on the large image datasets as the network backbone for feature extraction, and fine-tuning the pretrained models on the US data [31]. The discrepancy between the human experts in annotation may be another issue in tasks regarding the morphological measurement.

The objective of this study is to demonstrate the feasibility of implementation of DL models as clinical tools to aid an automated, objective visualization of MN boundaries in dynamic US acquired during finger flexion and extension. Our primary interest is to evaluate the feasibility of using several state-of-the-art DL models to effectively segment the MN in dynamic US. Both semantic and instance segmentation were employed for comparison. The former directly localizes MN by enclosing all pixels classified as the nerve in an image, while the latter localizes many MN candidates in the image and associates each candidate with a prediction confidence score. Since there is only one MN in individual frame, the candidate with the highest confidence score was chosen to represent the MN in the frame. This strategy was anticipated to prevent from the erroneous segmentation of more than one nerve in the input image, an issue frequently encountered in semantic segmentation. In the present work, DeepLabv3+ [28] and Mask R-CNN [32] were chosen for the main semantic and instance segmentation approach, respectively, because both were ranked as the top-performing architectures during our subject recruitment. Other modules chosen for performance comparison were U-Net and feature pyramid network (FPN). U-Net has been widely utilized for nerve segmentation, while FPN is reported to achieve results comparable with that of DeepLabv3+ but more computationally efficient for feature extraction of high resolution [33,34]. U-Net and DeepLabv3+ correspond to the two structures popular in semantic segmentation, symmetric encoder-decoder and atrous convolution, respectively. A few ablations including variation of model backbone, size of input image and output stride, were conducted to determine the balance between inference speed and segmentation effectiveness. Transfer learning and fine-tuning were applied with model backbones pretrained on ImageNet and coco_2017_train dataset. Our secondary interest is to compare the morphological features of the MN annotated by one human expert with that inferred by the models trained with datasets labeled by another expert. The features retrieved from the images included the spatiotemporal profile of nerve centroid, the changes in nerve circularity, and the changes in nerve CSA, during finger motions. We found that, both DeepLabV3+ and Mask R-CNN pretrained on ImageNet and coco dataset predicted MN with the score of intersection over union around 83% in average, and the morphological features of the MN exhibited the minimal discrepancy in the spatiotemporal profile of the nerve centroid between those predicted by the models and annotated by another expert. Our works evaluated the effectiveness of MN segmentation across a variety of the state-of-the-art DL models, and successfully demonstrated the feasibility of automatic extraction of the morphological dynamics of the MN during fingers motion using these models. To our best knowledge, our work was the first time to demonstrate the common pattern of the morphological dynamics extracted by the DL models, and our data suggested that the spatiotemporal profile of the nerve centroid may exhibit the most consistent behaviors when comparing the morphological dynamics of MN acquired from the subjects of similar population but annotated by different investigators. Our works highlight the potentiality of incorporating these models into an US machine to reduce the burden of clinicians in the acquisition of the morphological dynamic of MN in dynamic US, which may facilitate the disclosure of additional MN features characterized in CTS.

## 2. Materials and Methods

### 2.1. Model Principles

Two techniques in automatic image annotation greatly benefitted by DL are semantic and instance segmentation. Semantic segmentation assigns each pixel in image a class label according to the object within which the pixel is enclosed, and all objects of the same class are grouped as one entity. On the other hand, instance segmentation goes a step further and assigns individual detected object a distinct instance. The DL models employed in the present work were U-Net, DeepLabv3+, and FPN for semantic segmentation, and Mask R-CNN for instance segmentation, respectively. The main principle of the four models is briefed in the following.

#### 2.1.1. U-Net

Fully convolutional network (FCN), the pioneer of DL-based semantic segmentation methods, replaces the fully connected layers in CNN by convolutional layers to classify individual pixel [35]. Since the task of segmentation mainly involves the classification of individual pixels, the spatial information at pixel level is important. The so-called skip connection combining the semantic information retrieved in the higher layers with the intermediate output rich in spatial cues from the lower layers is employed, to reduce the progressive loss of feature resolution in the former, a main issue when the structure increases depth. The follower U-Net [27] proposed an encoder-decoder structure to obtain better semantic information with preserved spatial information. The encoder path down--samples the input image by successive pooling operation and convolution striding to extract semantic information, while the decoder path progressively up-samples and combines high-level features with the low-level ones provided by the encoder path. The decoder path is symmetric to the encoder one and lead to a symmetric u-shaped architecture. In addition to nerve segmentation, the FCN and U-Net based structures had been widely employed in the automatic segmentation of a variety of anatomical structures in US, including liver [36], breast [37,38], and thyroid [39].

#### 2.1.2. DeepLabv3+

An alternative to the encoder-decoder structure is atrous (dilated) convolution [40], which replaces the convolution striding by dilating the kernel scale to broaden the receptive field. Irfan et al. applied atrous convolution to extract semantic feature for breast lesion in US, with preservation of the spatial information during the progression of feature extraction [41]. Atrous convolution-based methods have recently dominated the leaderboards of semantic segmentation models, and DeepLabv3 is one of the top-ranked architectures [42]. DeepLabv3 consists of an improved atrous spatial pyramid pooling (ASPP) module [43] that employs atrous convolution of different dilation rates in parallel to capture the object features at multiple scales, as well as an average pooling to extract the global features, and the results are concatenated together to yield the last feature map. DeepLabv3+ [28], an improved version of DeepLabv3, combines the ASPP module with an encoder-decoder structure. With DeepLabv3 being the encoder, DeepLabv3+ adopts a simple decoder module that utilizes skip connection with the low-level features to obtain sharper object boundaries. Flores et al. recently compared the segmentation performance of breast tumor in US using various DL models and shows that the results obtained by DeepLabV3+ were slightly better than that of U-Net [44].

#### 2.1.3. FPN

Although the application of dilated convolution effectively improves feature resolution, the complex architecture also increases the cost of computation time and memory, which limits the choice of backbone network and the size of input image. FPN was originally designed for fast object detection [34]. Unlike U-Net, FPN is characterized by an asymmetric, lightweight decoder consisting of single block in individual stage and shared channel dimension. Kirillov et al. added a lightweight dense-prediction branch on top of FPN, called semantic FPN, to enable pixelwise classification [33]. Semantic FPN has been shown impressive results in semantic segmentation comparable with the top-performing dilation-based systems such as DeepLabV3+ on Cityscapes dataset [33,45]. Wu et al. recently proposed a FPN-based model enhanced by a boundary-guided feature enhancement module to improve the segmentation of breast lesion in challenging US datasets [46].

#### 2.1.4. Mask R-CNN

As for instance segmentation, one of the most successful models is Mask R-CNN (regions with CNN features), which involves localizing individual region of object with a bounding box, followed by the object classification and segmentation [32]. In addition to CNN, Mask R-CNN utilizes FPN to enhance extraction of feature of different scales. A region proposal network scans through the feature map of various levels to propose region candidate of objects enclosed by bounding boxes. Object classifier and FCN module are then employed in parallel for each proposed region to tune the mask segmenting individual objects. Mask R-CNN has been employed for the automatic segmentation of the breast tumors on sonograms with a mean average precision of 0.75 [47].

### 2.2. Subject Recruitment and Dataset of Dynamic US Images

Institutional review board approval was obtained for this prospective study (IRB No. NTUH-REC 201711014RINA). All participants provided written informed consent. Inclusion criteria were (1) aged 20–80 years; (2) diagnosed with idiopathic CTS according to clinical and electrodiagnostic criteria [48,49]. Exclusion criteria were histories of wrist surgery, traumatic wrist injury within 2 years, previous wrist injection within 3 months, history of peripheral nerve injuries (brachial plexopathy, cervical radiculopathy or thoracic outlet syndrome), history of thyroid or autoimmune disease, and inability to cooperate with study protocol. Subjects exhibited bifid MN were excluded in the present work since this type of MN morphology is rarely seen. There were totally 52 subjects enrolled in this study from 2018 to 2019. Dynamic US images of MN were acquired by one physiatrist with 3-year experience of musculoskeletal US, using a 13–18 MHz linear transducer (Aplio 500, Canon Medical Systems Europe B.V., Zoetermeer, the Netherlands). All participants were positioned with the palm facing upward and the wrist in neutral position. The transducer was placed at the level of proximal inlet (between the pisiform and scaphoid bones), and MN was identified in the transverse view. The participants were instructed to perform neutral extension of their fingers initially, followed by full flexion (clenched-fist posture) and then back to finger extension (open-palm posture). The transducer was tilted accordingly during motions to avoid anisotropy of the MN. Such flexion-extension cycles were repeated for 5 times, lasting for 10–15 s. Active finger flexion and extension induce MN displacements, and the transverse MN sliding within the carpal tunnel can be clearly observed in US dynamic imaging. Video clips with 38 frames-per-second were recorded. Note that there might be more than one video acquired for a single subject. The videos of 16 subjects exhibiting a large variety of image features as determined by the expert were selected for the test dataset and excluded from the training procedure. The videos of the remaining 36 subjects were used for model training. Sequential images were picked at an interval of four or ten frames from the videos and the MN boundaries were manually labeled by another physiatrist with musculoskeletal US expertise using Labelme [50]. The training and test dataset consisted of 15,215 and 3410 frames, respectively. Before model training and test, each frame was transformed from RGB to gray scale to remove the visual enhancement effects provided by the US manufacturer. Data augmentation included random brightness and contrast adjustment, random resizing, and random left-right flipping, were applied to the training frames.

### 2.3. Model Implementation

The DL models chosen for semantic segmentation of MN in dynamic US were DeepLabv3+, U-Net, and the semantic FPN proposed in [33]. Mask R-CNN was chosen as our instance segmentation model and the object with the highest confidence score in each frame was as selected as MN. Each approach was trained for 465K iteration with batch size of 2. The learning rate was decreased by 10 at the 274K and 366K iteration. The training process for individual approach with various initial setting was conducted once. For DeepLabv3+, we followed the training protocol described in [28] and set the learning schedule as initial learning rate of 0.015, a weight decay of 0.0001, a momentum of 0.9, and frozen batch normalization parameters. The default output stride was set as 16 and the size of the input image was cropped into 721 pixels in height and 961 pixels in width due to constraint of GPU memory. For Mask R-CNN, we also followed the training protocol described in [51], except that the initial learning rates were changed to 0.002 because the size of our dataset was different than that of ImageNet and coco_2017_train. The values of the weight decay and the momentum were set as the same as that in DeepLabv3+. We trained U-Net and FPN with the same parameters as Mask R-CNN, except that the initial learning rate was set to be 0.1, since their architectures for feature extraction were similar to but simpler than that of Mask R-CNN. When the performance of the four models was compared, ResNet-101 initialized with weights pretrained on ImageNet was chosen as the main CNN backbone. In this context, the encoder path of U-Net was implemented with ResNet-101, and the output feature maps of the last layer of each stage were skip-connected to the decoder path. The channels of the feature maps were reduced by applying 1 × 1 convolution before being concatenated with the feature in the decoder path to maintain the structural symmetry. The effects of backbone variation on the model performance were evaluated by implementing DeepLabv3+ with modified aligned Xception-65 for image segmentation and ResNet-101 for feature extraction [28], as well as implementing U-Net and FPN with ResNext, which mixed the split-transform-merge idea from inception module into ResNet to improve feature extraction [52].

The performance of DeepLabv3+ and Mask-R-CNN was further compared by varying training settings. Both models were initialized with weights pretrained on coco_2017_train dataset and fine-tuned on the US data. We trained DeepLabv3+ with output Stride 8 and 16, respectively, by changing the atrous rate in the encoder path. Note that the atrous rates in ASPP were changed accordingly to retain the same designed scale of feature extraction, which were 12, 24, 36 for output Stride 8, and 6, 12, 18 for output Stride 16, respectively. Due to the constraint of GPU memory, the input images used for DeepLabv3+ model training with output Stride 8 were randomly cropped into 481 by 481 pixels in size, which was the maximum size allowed by our system at that output stride setting. To maintain the global and local spatial features, we performed multi-scale training for the DeepLabv3+ model by randomly resizing the US image by 0.5, 0.75, 1, 1.25, or 1.5 times at each iteration to augment data. The resized images were further cropped into a fixed size of 721 by 961 or 481 by 481 pixels for data input. Multi-scale training was also conducted in Mask R-CNN model implemented with ResNet-101 or ResNext as the backbone, with that the input images were randomly resized into 360, 540, 720, 900, or 1080 pixels in height in each mini epoch. Note that unlike DeepLabv3+, input images of arbitrary size in the training session of Mask R-CNN were allowed and image cropping was not required. In the present work, cross-validation was not performed, since our primary goal was to evaluate the feasibility of using these models to effectively segment MN in dynamic US, rather than the achievement of the best results by improving the hyper-parameters.

### 2.4. Morphology Metrics

The centroid position, circularity, perimeter, and CSA of the MN in individual frame were automatically determined using the binary mask that segmented the nerve from the image. Let the Cartesian coordinates *x* and *y* axes denote the radial-ulnar and palmar-dorsal directions on the image, respectively, and the numbers of pixel along the *x* and *y* direction are *w* and *h*, respectively. The MN CSA was calculated from the zeroth moment of the binary mask, namely

(1) $$\mathrm{CSA}=\overset{x=w}{\underset{x=0}{\sum }}\overset{y=h}{\underset{y=0}{\sum }}x^{0}y^{0}f\left(x,y\right)=\overset{x=w}{\underset{x=0}{\sum }}\overset{y=h}{\underset{y=0}{\sum }}f\left(x,y\right),$$

where $f\left(x,y\right)$ represents the values of the binary mask at pixel $\left(x,y\right)$, and is equal to 1 and 0 as the pixel is inside and outside the mask, respectively. Hence, the CSA is indeed the total number of pixels classified as the MN. The coordinates of the MN centroid, $\left(c_{x},\;c_{y}\right)$, was derived from the first moment of the mask along the *x* and *y* direction and expressed as

(2) $$c_{x}=\overset{x=w}{\underset{x=0}{\sum }}\overset{y=h}{\underset{y=0}{\sum }}x^{1}y^{0}f\left(x,y\right)/\mathrm{CSA}=\overset{x=w}{\underset{x=0}{\sum }}\overset{y=h}{\underset{y=0}{\sum }}xf\left(x,y\right)/\mathrm{CSA},$$

(3) $$c_{y}=\overset{x=w}{\underset{x=0}{\sum }}\overset{y=h}{\underset{y=0}{\sum }}x^{0}y^{1}f\left(x,y\right)/\mathrm{CSA}=\overset{x=w}{\underset{x=0}{\sum }}\overset{y=h}{\underset{y=0}{\sum }}yf\left(x,y\right)/\mathrm{CSA}.$$

The MN circularity was defined as

(4) $$circularity=4\pi \;\cdot \;\frac{\mathrm{CSA}}{p_{MN}^{2}}\;,$$

Which indicates the segmented MN resembled a circle if the value was equal to 1, and the smaller the value, the less roundness of the nerve shape. $p_{MN}$ denotes the perimeter of the segmented nerve and was calculated using the arcLength function in OpenCV. To facilitate comparison with the data reported in literature, the calculated values of centroid position, perimeter, and CSA were further converted into unit of cm based on the scale provided by the US images.

### 2.5. Performance Evaluation

The model performance was evaluated by calculating the values of the intersection over union (IoU) between the ground truth data labeled by the physiatrist and that predicted by the models for individual frames in the test video, as well as the inference time. To minimize the weighting effect resulting from the unbalanced number of the sampled frame in each video and highlight the difference among the videos, the effectiveness of MN segmentation using various models was evaluated per video, rather than individual frame. The inference accuracy of each video was represented by the variable average IoU, defined as the average of the IoU values of all the sampled frames in the video. Significance of the performance difference across various models was analyzed. The data normality was checked using the Kolmogorov–Smirnov and Lilliefors test. For normally distributed data, the significance of differences was evaluated by ANOVA with post hoc Tukey’s test, whereas the Kruskal–Wallis test were employed for non-normally distributed data. A *p*-value less than 0.05 was considered significant. The inference time was calculated by averaging the time spent for MN prediction in individual images across the video. All the computation was conducted using chips of NVIDIA GEFORCE GTX 1080 Ti.

## 3. Results

### 3.1. Effects of Model and Backbone Variation on Inference Performance

We first evaluated the performance of various models implemented with different backbones in MN segmentation, as summarized in Table 1. The effectiveness of the MN segmentation in each test video was quantified by the average IoU and there were totally 81 videos included in the test set. It appears that the highest score was achieved by DeepLabv3+ implemented with Xception-65, immediately followed by Mask R-CNN implemented with ResNet-101, although the difference of the average IoU scores across different models was not statistically significant. The fastest inference speed was achieved by FPN implemented with ResNet-101, which was roughly 0.05 s per frame or 20 frame per second (FPS). Improvement in the average IoU score were also seen in U-Net and FPN model when the backbone was changed from ResNet-101 to ResNext-101-32x8d, although the scores still fell behind that of DeepLabv3+ and Mask-R-CNN. The 32x8d notation indicates that the backbone consisted of 32 groups of residual transformation blocks of the same topology (cardinality = 32) at the second stage, with that the channel number of individual groups was 8. These results agree well with the fact that ResNext-101-32x8d defeats ResNet-101 in the ImageNet classification dataset and is considered to be better in feature extraction. The tradeoff, however, is the substantial increase of the computation time, which was roughly doubled when the backbone was changed. As for DeepLabv3+, changing the backbone into Xception-65 did improve the IoU score without considerable increase of computation time. Note that the model was trained with output Stride 16 and without cropping of the input image, and tested with output Stride 16 in both backbone conditions. These results indicate that DeepLabv3+ and Mask R-CNN are capable of providing MN automatic segmentation with considerable accuracy at an inference speed approximately of 10 FPS.

### 3.2. Effects of Various Training Conditions on Model Performance

Given that the top-ranked IoU scores in MN segmentation were achieved by DeepLabv3+ and Mask R-CNN, we then leveraged the performance of these two models with various training conditions. Table 2 lists changes in the performance of DeepLabv3+ model trained in conditions with various output strides, with and without cropping of the input image, and in that of DeepLabv3+ and Mask R-CNN trained with and without the presence of multi-scale input. Almost all of the resultant values of averaged IoU exceed 0.82. Although the difference of the IoU scores across various conditions was not statistically significant, the highest score occurred when the MN was predicted by DeepLabv3+ trained with smaller output stride, multi-scale input, and cropping of the input image. For both models trained with various settings, the variation of the IoU scores across all test videos was around 0.05–0.06, roughly 7% of the averaged value. Note that the number of output stride is referred to as the scale ratio of the input image to the feature map after down-sampling. Hence, a smaller output stride was expected to yield a high-level feature map of higher resolution, yet cost more computation time. However, halving the output stride number did not substantially improve the IoU score in our dataset. Training with multi-scale input improved the IoU score in both DeepLabv3+ and Mask R-CNN models, irrespective of whether the input image was cropped or not in the former. This is reasonable because multi-scale input enriched the training dataset by providing MN image of a variety of size, which prevented from imbalanced training of a particular level for feature extraction when the MN size in the input image was out of the size range processed by the level. As mentioned earlier, we were forced to randomly crop the input image into a smaller size to meet the constraint of GPU memory when the model was trained with smaller output stride. Although the size of the cropped image was still larger than that of MN, we thought that the random cropping may impede the learning efficacy if part of the MN was cropped from the input image. However, training with cropped image did not always yield lower accuracy in MN prediction, which is against what we assumed. These results suggest that, as for our dataset, training DeepLabv3+ with local features may be more beneficial than with global features. In general, the echotexture of MN exhibits a so-called honeycomb appearance which is characterized by a collection of hypoechoic fascicles interspersed with a meshwork of echogenic perineurium and the whole structure is enclosed by a hyperechoic outer lining (the epineurium). Image cropping may force the model to learn to detect MN primarily based on the local feature (i.e., the honeycomb appearance), rather than a structure surrounded by an intact hyperechoic outer lining, if the MN was partially cropped from the input image. As for Mask R-CNN model, change of backbone from ResNet-101 to ResNext-101-32x8d alone doubled the computation time without substantially improving the IoU score. Taken together, these data highlight the importance of multi-scale input to improve the inference accuracy in both models.

### 3.3. Conditions Affect the Model Inference

**Figure 1:**
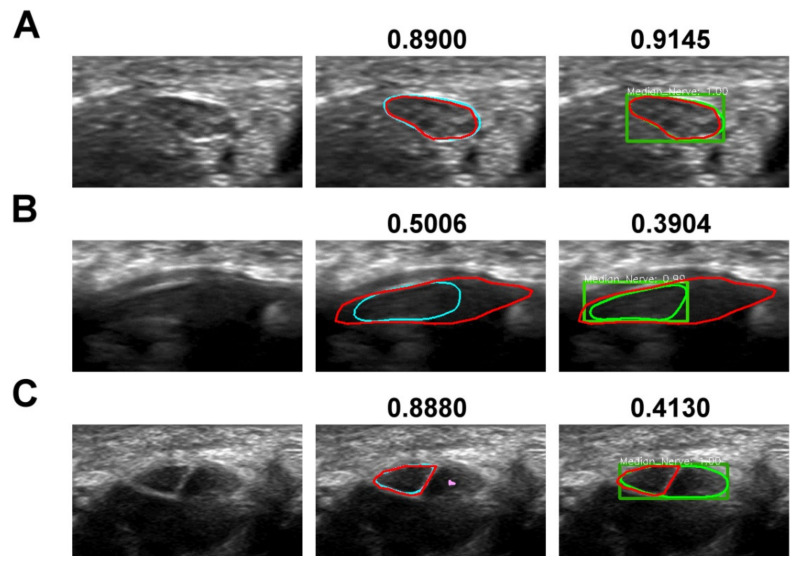
MN US images with various IoU scores. The first, second, and third column refers to the input image, and the image superimposed with MN region predicted by DeepLabv3+ and Mask R-CNN, respectively. The red, light blue, and light green line represents the ground truth, and the segmentation of MN predicted by DeepLabv3+ and Mask R-CNN, respectively. The dark green rectangle denoted the bounding box of the highest confidence score for MN in Mask R-CNN. The numbers above individual panel denoted the corresponding IoU scores. (**A**) MN image with high IoU scores in both models; (**B**) MN with blurred appearance and low IoU scores in both models; (**C**) MN image with clear but ambiguous appearance. The MN predicted by DeepLabv3+ with the largest area successfully recapitulated the true MN region, and that of a smaller size (labelled with pink color in the hypoechoic structure) was excluded from the final output. However, the MN segmented by Mask R-CNN mistook the hypoechoic structure for part of the nerve.

Given that there was roughly 7% variation of the IoU values across the test videos in both models, we wondered whether the IoU score was dependent on the quality of the input images. Inspecting individual test frames and the associated IoU scores revealed that the low IoU scores mainly arose from two image conditions, a MN with blurred appearance, and a MN with clear yet ambiguous pattern. Figure 1 exhibits examples of segmented MN with high and low IoU scores owing to the aforementioned conditions. The MN enclosed by the light blue line was predicted by DeepLabv3+ trained with multi-scale input, backbone of Xception-65, training crop size of 481 by 481 pixel, and output Stride 8, whereas that delineated by the light green line was predicted by Mask R-CNN implemented with ResNext-101-32x8d and trained with multi-scale input, with that the dark green rectangle represented the bounding box of the highest confidence score. The true MN region determined by the experts was marked with the red line. The numbers above individual panel denoted the corresponding IoU score of the particular frame. In Figure 1A, both segmentation methods yielded high IoU scores, and the predicted MN regions were almost the same as that enclosed by the experts. In Figure 1B, the MN appeared blurred, probably owing to that the image was not sampled fast enough to catch the moved MN, and the MN predicted by both models only partially matched that localized by the experts.

Figure 1C demonstrates an issue commonly encountered by all segmentation-based methods. The MN appeared clearly defined, but there was a hypoechoic structure (marked by the pink spot) adjacent to the MN and the two structures may be mistaken for a fragmented MN by inexpert specialists. The hypoechoic structure was indeed part of the flexor digitorum tendons, which are normally positioned next to the MN. Since semantic segmentation is aimed to perform pixelwise classification, the hypoechoic structure was erroneously labeled by DeepLabv3+ as another instance of MN of tiny size (labelled by pink color). However, we solved this problem by enforcing the model to choose the segmented region of the largest area as the MN, which successfully recapitulated the true MN region. In contrast, the trained Mask R-CNN model mistook the two structures for the MN. This discrepancy probably resulted from the difference in spatial resolution between the high-level feature map extracted by the two models. The feature map of the highest level generated by Mask R-CNN had an output stride equivalent to 32, which was far larger than that set in DeepLabv3+ and may be less robust to differentiate local structure of ambiguity. Thus, erroneous prediction occurred once the proposed bounding box mapped to the feature map of the highest level obtained the highest confidence score.

### 3.4. Morphological Characteristics of the Inferred Median Nerve

**Figure 2:**
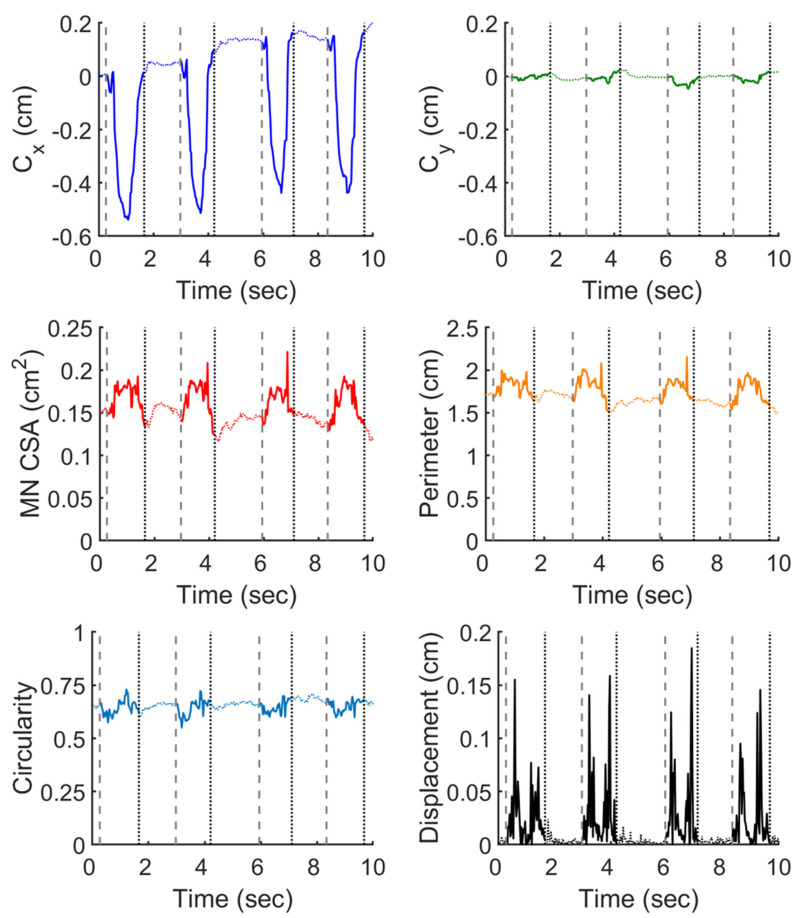
Typical temporal profiles of the morphological characteristics of an inferred median nerve (MN) during fingers motion, including the *x* (*C*x) and *y* (*C*y) coordinates of the MN centroid, the cross-sectional area (CSA) and the perimeter of the MN, the MN circularity, and the centroid displacement between consecutive images. The data were grouped by four cycles of finger flexion and extension, with that the vertical gray dash lines represent the beginning of each cycle and the black dotted lines denote the end of the cycle. Data collected within and out of individual cycles were depicted in solid and dotted line, respectively.

Of great interest to most clinician in the application of the DL-based models is to automatically retrieve several morphological features from the inferred nerve to assist clinical diagnosis and management. The morphological features addressed in the present work were the temporal profile of the centroid position of the inferred MN, and the temporal variation of the MN circularity, CSA, and perimeter, during the finger motions. Figure 2 demonstrated an example of these profiles traced from the inferred MN during four successive motions of fingers flexion and extension of one subject. During the finger motion cycle, the MN centroid exhibited larger displacement in *x* direction (positive for scaphoid side) than that in *y* direction (positive for palmar side) and moved primarily toward the negative *x* direction; the CSA and perimeter was roughly enlarged by 20% and 10% during finger flexion, respectively, and shrunk back when fingers extended; the nerve became less circular during finger flexion and recoiled as fingers extended; and the centroid displacement exhibits a biphasic pattern, corresponding to the flexion and extension of the fingers.

**Figure 3:**
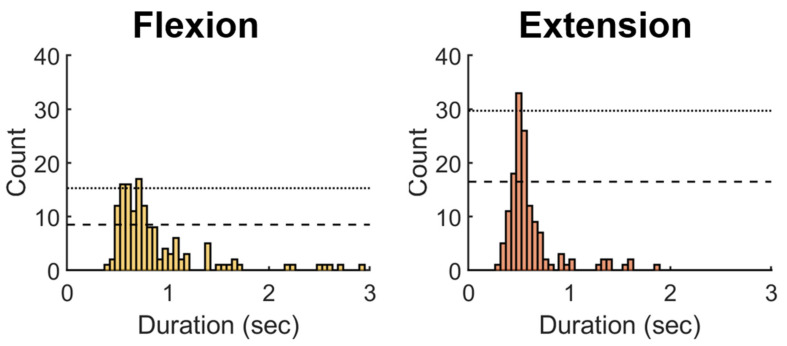
Histogram of the time spent for fingers flexion and extension that were estimated from the inferred images. The dotted and dash line represented the 90% and 50% value of the maximum count, respectively. The duration ranges include the bars of count larger than or equal to the 90% and 50% value were defined as D_90_ and D_50_, respectively.

We then examined the common pattern of these features by pooling the data derived from the MN inferred in the test dataset. Data associated the finger flexion and extension phase in individual motion cycle were manually separated based on the biphasic pattern of the centroid displacement as shown in Figure 2. Figure 3 depicts the histogram of the time spent for the individual finger flexion and extension phase extracted from the motion cycle, respectively. The duration of the finger flexion and extension that occurred the most frequently was around 0.68–0.71 and 0.47–0.5 s, respectively; namely the whole motion cycle lasted slightly over 1 sec. The dotted and dash line shown in Figure 3 represented the 90% and 50% value of the maximum count (frequency). We defined D_90_ and D_50_ as the duration ranges with frequency larger than or equal to the 90% and 50% value of the maximum, respectively. Hence, the D_90_ and D_50_ were 0.52–0.71 and 0.47–0.87 s for the flexion, and 0.47–0.5 and 0.42–0.55 s for the extension phase, respectively. The varied duration of individual phases rendered it difficult to profile the common pattern against time simply by aligning the data sequence of individual phase with respect to the phase beginning and averaging them at each time point. To facilitate alignment of the temporal sequence, we normalized the phase durations of similar length with respect to themselves and resampled the data at an interval of 1/29 relative to the normalized duration. In other words, there were totally 30 data points resampled at a fixed interval throughout the collected data sequence. The resampled data sequences were temporally aligned, averaged, and profiled against the normalized duration.

**Figure 4:**
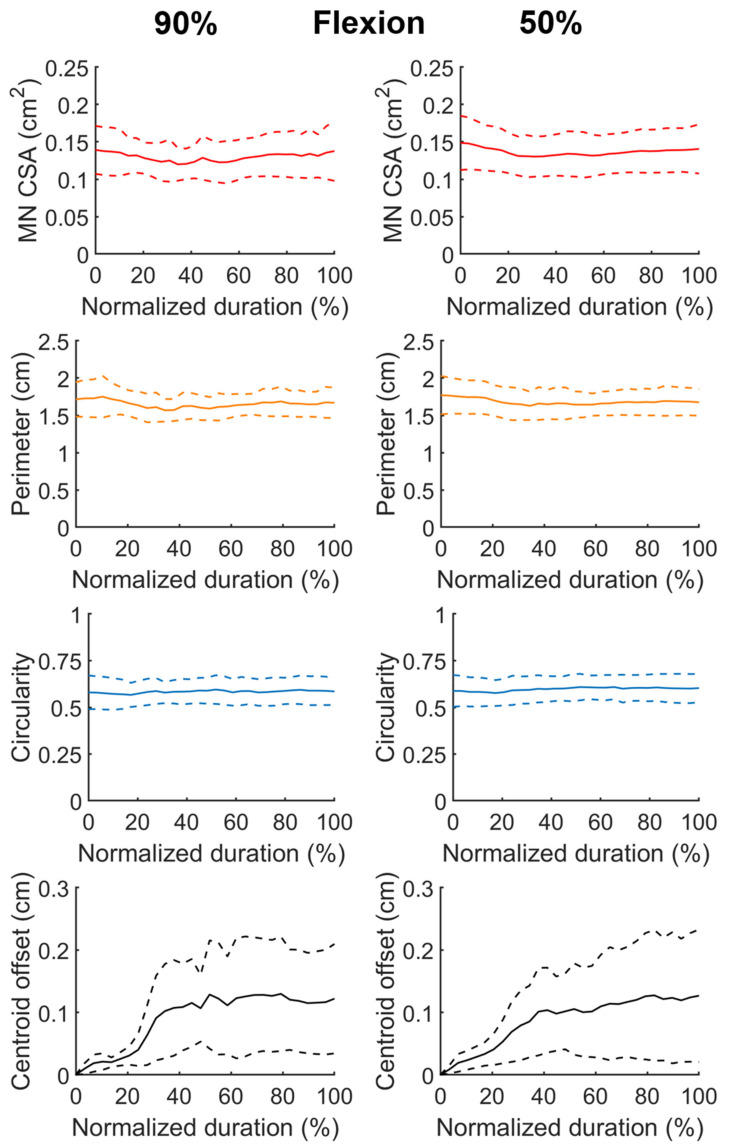
Temporal profiles of the morphological characteristics of inferred MNs during fingers flexion, including the CSA and the perimeter of the nerve, the MN circularity, and the centroid displacement throughout the motion. The phase duration was rescaled by normalization. The data shown in the left and right column were pooled from the data of duration confined by the D_90_ and D_50_ defined in Figure 3, respectively. In individual panel, the solid line represents the average of the resampled data, and the dash lines stand for average plus and minus one standard deviation, respectively.

**Figure 5:**
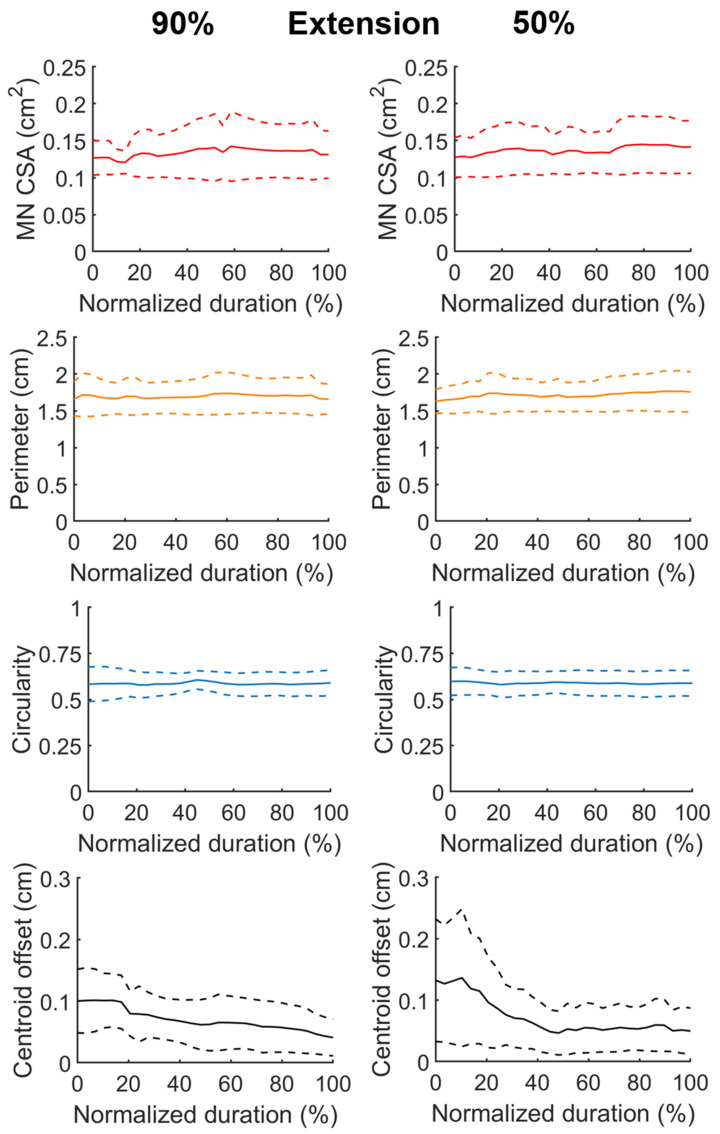
Temporal profiles of the morphological characteristics of inferred MNs during fingers extension, including the CSA and the perimeter of the nerve, the MN circularity, and the centroid displacement throughout the motion. The phase duration was rescaled by normalization. The data shown in the left and right columns were pooled from the data of duration confined by the D_90_ and D_50_ defined in Figure 3, respectively. In the individual panel, the solid line represents the average of the resampled data, and the dash lines stand for average plus and minus one standard deviation, respectively.

Figure 4 and Figure 5 depicted the temporal profiles of the MN CSA, perimeter, circularity, and centroid offset with respect to the normalized duration in finger flexion and extension phase, respectively. The centroid offset represented the spatial deviation relative to the centroid position at the beginning of finger flexion phase. At each normalized time point, the data were represented by the average (the solid line) and that plus and minus one standard deviation (the dash lines). In both Figure 4 and Figure 5, the data shown in the left and right column were collected from the phases of duration confined by D_90_ and D_50_, respectively. In general, the data shown in the left and right column followed a similar trend with that the latter appeared smoother owing to their larger sample size. The MN CSA was shrunk about 12% in average when finger flexed and recoiled as finger extended. The change in the nerve perimeter during finger motion was in accordance with that of CSA. The nerve circularity varied minimally throughout the motion cycle and the value was approximately 0.6, which indicates that most of the time the nerve shape appeared as an equilateral triangle. The nerve centroid was displaced approximately 1 mm in average in a reversible manner during the motion, with that the majority of displacement occurred within the 40% duration from the initiation of the flexion and extension phase.

**Figure 6:**
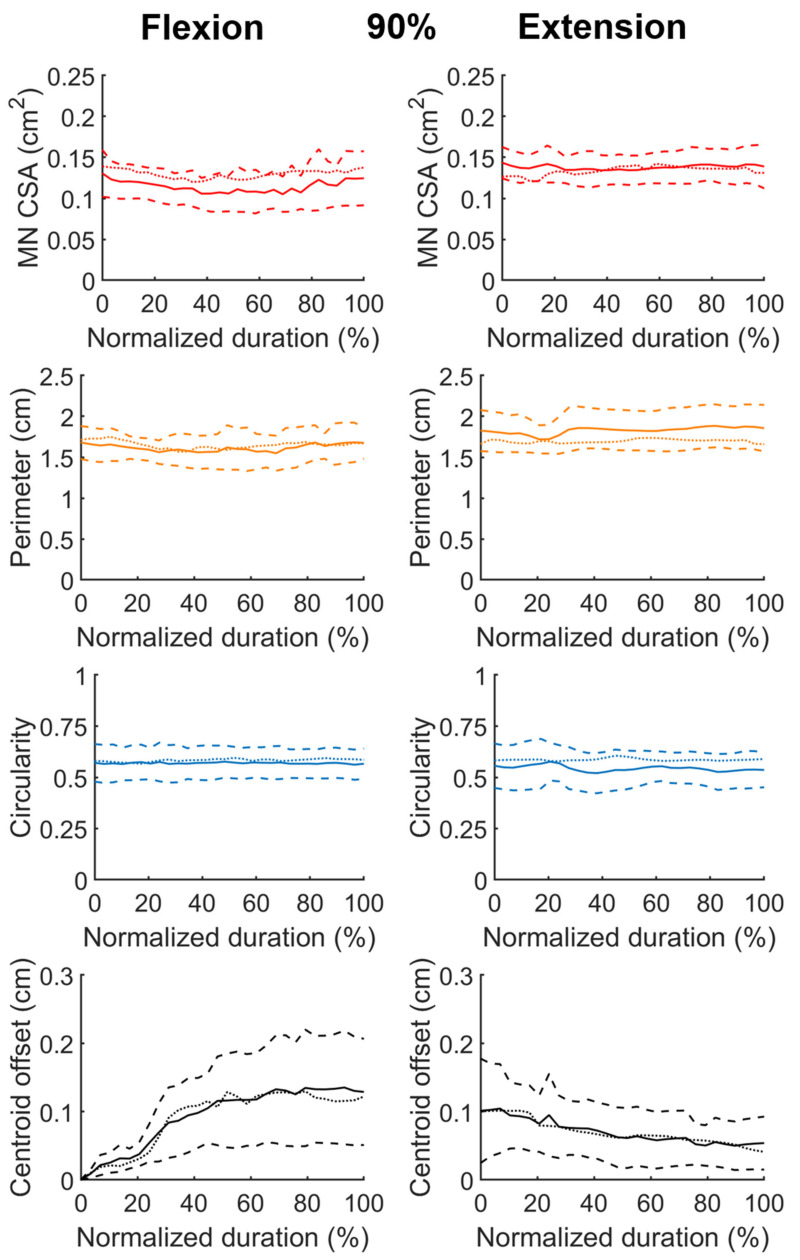
Temporal profiles of the morphological characteristics of MNs labelled manually during fingers flexion and extension, including the CSA and the perimeter of the nerve, the MN circularity, and the centroid displacement throughout the motion. The phase duration was rescaled by normalization. The data were pooled from the data of duration confined by the D_90_ defined in Figure 3. In the individual panel, the solid line represents the average of the resampled data, the dash lines stand for average plus and minus one standard deviation, and the dotted line refers to the average calculated from the inference data, respectively.

We finally examined whether the inference results met the clinical consensus. We compared the morphological features calculated from the inference data with that manually measured from the same image sequences by another expert who did not participate the labeling of the ground truth in the model training. Figure 6 exhibits the comparison using data collected from the phases of duration confined by D_90_ shown in Figure 3. It appears that the inference data agree well with that measured manually in the MN perimeter and circularity during finger flexion, the MN CSA during finger extension, and the centroid displacement throughout the motion. In contrast, the inferred MN CSA exceeded the manually measured ones by approximately 13% in average during finger flexion; during finger extension, the inferred nerve perimeter was shorter than the manually measured ones by approximately 7% in average, and the average discrepancy between the inferred and manually measured circularity was around 8%. These discrepancies may be attributed to the errors in model prediction, given that the best IoU values delivered by our model were less than 0.84. Besides, the data disagreement may arise from the subjective variation across observers in defining the MN boundaries. If the difference in boundary definition was consistent throughout the nerve peripheries, it was expectable that there would be the least difference in the centroid displacement throughout the finger motion, since the centroid positions would vary insignificantly when the boundaries were homogeneously enlarged or contracted.

## 4. Discussion

In the present work, MN was automatically delineated and traced across US image sequences acquired from CTS patients in a robust manner using DL-based segmentation models. The averaged IoU score exceeded 0.83 when the model was DeepLabv3+ implemented with backbone of Xception-65, and trained with multi-scale input, cropped input images, and output Stride 8, and Mask R-CNN implemented with ResNext-101-32x8d as backbone and trained with multi-scale input. When fingers actively flexed and then extended, the inferred MN was displaced preferentially toward the ulnar side only for 1 mm in average and then back to the radial side; the MN CSA was slightly shrunk by about 12% in average in finger flexion and swelled back as finger extended, with that over 84% (data range above mean minus one standard deviation) of the estimated CSA was larger than 0.1 cm^2^ throughout the motion; accordingly, the nerve perimeter was shortened by about 8% when finger flexed and recoiled back during finger extension; the nerve circularity changed minimally and the nerve appeared as an equilateral triangle most of the time throughout the motion. These findings were consistent with those reported previously [9,11,12].

US has been regarded as a useful tool for CTS diagnosis in conjunction with NCS. Proposed diagnosis criteria for CTS using US include MN enlargement at carpal inlet, as well as limited MN deformation in terms of circularity and reduced MN displacement during finger and wrist motions [11,12,13]. However, the manual recognition and delineation of MN in consecutive images require massive human labors. Therefore, the most popular approach is to analyze the MN in the initial and final frames of the selected motion, which ignores the temporal variation of the nerve pattern across the motion and may overlook features potentially correlated with the disease progression and severity. In the current study, we successfully used DL-based models for automatic segmentation of MN in dynamic US. Our work may have potential to be incorporated into a US machine to perform real-time MN segmentation, and automatically estimate MN CSA and displacement during finger motion. This will allow clinicians to acquire the dynamic information regarding MN morphology and movement more efficiently. Furthermore, the automatic detection of the temporal changes in MN CSA, circularity, and displacement during finger and wrist motion may facilitate the disclosure of additional MN features characterized in CTS. Improved diagnosis and prediction of disease severity of CTS may be achievable by using DL models to handle the high-dimensional datasets containing NCS data and the rich temporal information provided by dynamic US. Ali et al. recently reported a framework utilizing ensemble deep learning and feature fusion approaches to handle data of different sources for heart disease prediction, and the reported accuracy was much higher than existing systems [53].

However, there is still plenty of room for improvement in the presented approaches. The images were acquired by one physician using a particular probe of one US machine. This implies that the models might be overfitted for the features exclusively provided by the training dataset, and less effective in the segmentation of US data acquired by other physicians or other US machines. Thus, expanding the training dataset with diverse sources is required to improve the model practicability. The inference speed of the DeepLabv3+ and Mask R-CNN model with the highest IoU score was approximately 2 and 6 FPS, respectively, which was far slower than that accustomed by most clinician in clinical operation and inappropriate for real-time analysis in its present state. Given that the blooming development of DL in recent years, improvement of inference speed with high precision is expected in the future by adopting updated, less complicated architectures combined with upgrades of the hardware. For example, Wang et al., recently reported the SOLO architecture simply consisting of an instance mask branch and a semantic category branch to accelerate the end to end process for instance segmentation. SOLO was reported to possess accuracy on par with Mask R-CNN yet double inference speed [54]. Srinivasu et al. recently proposed a MobileNet V2-based model that successfully classified skin diseases with minimal computational efforts [55]. Another issue encountered by both the semantic and instance segmentation approaches employed in the present work is the handling of multiple, disconnected regions labeled as MN. Semantic segmentation labels individual pixel with a particular class. Ideally, a well-trained model for semantic segmentation should learn that all the pixels classified as MN were locally connected, and herein that there existed globally only one entity of MN in one image. However, our model occasionally segmented several disconnected blocks as MN in one image in the test video. We fixed this by simply assigning the segmented region of the largest area as the MN, which appeared to work well in our dataset. However, erroneous inference was anticipated to arise when the correct segmentation did not possess the largest area, there existed more than one segmentation of the largest area, and the MN was segmented by several disconnected regions yet only the part with the largest area was chosen as the nerve. The similar segmentation issues may occur in the instance segmentation approach except that the disconnected segmentations were confined in the bounding boxes. Again, we solved this issue by simply choosing the segmented block of the largest area from the box of the highest confidence score. Because the scoring was part of the model output and trainable, we think this may make Mask R-CNN less erroneous in handling the multiple, disconnected segmentations issue when compared with DeepLabv3+. Nevertheless, the best way to eliminate these segmentation issues encountered in both approaches is to improve the segmentation accuracy. Given the video format of our dataset, this may be achievable by revising the model to adopt the information in time domain for MN segmentation, which is similar to the strategy that experienced physiatrists typically employ to deal with MN with blurred or ambiguous appearance. In general, they wound check the frames preceding and following the doubtful one to clarify the nerve morphology. Note that in the present work, we did not choose frameworks inherently advantageous for modeling sequence data, such as recurrent neural networks [56], since there is only one MN in individual frame and it was unnecessary to differentiate the object entity using the data sequence as required in tracing object with multiple instances in one image yet of similar morphology. Other reasons drove us to train the model on the basis of individual frame rather than the frame sequence included that the former approach cost less computation time and memory, and it was trained with a dataset of larger size, because it is obviously that the number of frames much outnumbered that of videos.

Dataset imbalance with respect to various size scale of MN is also an issue. Mask R-CNN adopts the FPN structure to improve the recognition of objects at different scales, which is primarily achieved by selecting the feature map of a particular pyramid level associated with the size of the proposed object candidate as the input for training of the mask segmenting the object [34]. Consequently, the training update of the model based on the detector loss generated in the stage of object classification, bounding box regression and segmentation, would be mainly conducted in paths connected with the selected feature map through back propagation. However, incomplete training for the detection of a particular class may occur if the training data of the class exhibit unbalanced distribution across various scales. In our dataset, over 95% of the MN CSA were within the range of 56 by 56 to 112 by 112 pixels, which corresponds to the feature map at pyramid Level 4 (P_4_). This alludes that paths connected with feature maps at level other than P_4_ were trained with data less than 5% of the total. Given that there was no constraint imposed on MN CSA in the present work, the insufficient training of the paths not connected with feature map P_4_ may lead to less accurate model prediction when the MN CSA was out of that of the 95%. We overcame the unbalanced distribution of objects scales by simply augmenting the data with randomly resizing. Since the MN CSA of normal subjects is expected to be smaller than that of CTS patients, increasing the diversity of the training dataset by including that of normal subjects in the future may be beneficial for solving this issue. Another potential approach is to increase the number of the minority cases in the dataset by over-sampling techniques, as recently proposed by Ijaz et al. for prediction of a variety of diseases, including cervical cancer, diabetes, and hypertension [57,58].

## 5. Conclusions

In summary, we successfully demonstrated the feasibility of automatically segmenting MN in dynamic US using DL-based models. The inference effectiveness across a variety of the state-of-the-art DL models was evaluated, whereas both DeepLabV3+ and Mask R-CNN pretrained on coco dataset predicted MN with IoU scores around 0.83 in average. The morphological dynamics of the MN during fingers motion extracted by the models was consistent with that reported in literature. The morphological features of the MN exhibited the minimal discrepancy in the spatiotemporal profile of the nerve centroid when those predicted by the models and annotated by another expert were compared. This finding implicates that the dynamic of the nerve centroid may exhibit the most consistent behaviors when comparing the morphological patterns of MN acquired across a variety of investigations. Our works highlight the potentiality of utilizing DL-based approaches as clinical tools with minimal clinician labor-demanding for objective, real-time diagnosis of CTS, and the possibility of revealing additional dynamic US features of CTS.

## Figures and Tables

**Table 1:** Performance of MN segmentation using different models with varied backbone.

Model	Backbone	Segmentation Type	Average IoU	Average Inference Time (s)
U-Net	ResNet-101	Semantic	0.7873 ± 0.0882	0.0623
U-Net	ResNext-101-32x8d	Semantic	0.8031 ± 0.0668	0.1239
FPN	ResNet-101	Semantic	0.8016 ± 0.0647	0.0556
FPN	ResNext-101-32x8d	Semantic	0.8132 ± 0.0608	0.1146
DeepLabv3+	ResNet-101	Semantic	0.8045 ± 0.0628	0.0794
DeepLabv3+	Xception-65	Semantic	0.8243 ± 0.0527	0.0984
Mask R-CNN	ResNet-101	Instance	0.8216 ± 0.0564	0.0849

**Table 2:** Model performance with various training conditions.

Model	Backbone	Training Output Stride	Test Output Stride	Training Image Size	Multi-Scale Input	Average IoU	Average Inference Time (s)
DeepLabv3+	Xception-65	16	16	721,961	No	0.8249 ± 0.0533	0.0982
DeepLabv3+	Xception-65	16	8	721,961	No	0.8278 ± 0.0508	0.3651
DeepLabv3+	Xception-65	16	16	481,481	No	0.8247 ± 0.0569	0.1002
DeepLabv3+	Xception-65	16	8	481,481	No	0.8244 ± 0.0513	0.3539
DeepLabv3+	Xception-65	8	8	481,481	No	0.8179 ± 0.0673	0.3644
DeepLabv3+	Xception-65	16	16	721,961	Yes	0.8315 ± 0.0562	0.1018
DeepLabv3+	Xception-65	16	8	721,961	Yes	0.8285 ± 0.0533	0.3700
DeepLabv3+	Xception-65	16	16	481,481	Yes	0.8342 ± 0.0480	0.1024
DeepLabv3+	Xception-65	16	8	481,481	Yes	0.8283 ± 0.0454	0.3706
DeepLabv3+	Xception-65	8	8	481,481	Yes	0.8356 ± 0.0481	0.3718
Mask R-CNN	ResNet-101				No	0.8242 ± 0.0541	0.0845
Mask R-CNN	ResNet-101				Yes	0.8317 ± 0.0555	0.0846
Mask R-CNN	ResNext-101-32x8d				No	0.8252 ± 0.0580	0.1525
Mask R-CNN	ResNext-101-32x8d				Yes	0.8300 ± 0.0570	0.1536

## Data Availability

The source codes for the models presented in this study are openly available in https://github.com/yl732/MedianNerveDL (accessed on 13 September 2021). The image data are not publicly available due to legal and privacy issues.

## Abbreviation

CNN, convolutional neural network; CSA, cross-sectional area; CTS, carpal tunnel syndrome; DL, deep-learning; FCN, fully convolutional network; FPN, feature pyramid network; IoU, intersection over union; MN, median nerve; NCS, nerve conduction study; US, ultrasonography.

## References

[1] Alfonso C., Jann S., Massa R., Torreggiani A. (2010). Diagnosis, treatment and follow-up of the carpal tunnel syndrome: A review. Neurol. Sci..

[2] Dale A.M., Harris-Adamson C., Rempel D., Gerr F., Hegmann K., Silverstein B., Burt S., Garg A., Kapellusch J., Merlino L. (2013). Prevalence and incidence of carpal tunnel syndrome in US working populations: Pooled analysis of six prospective studies. Scand. J. Work. Environ. Health.

[3] Witt J.C., Hentz J.G., Stevens J.C. (2004). Carpal tunnel syndrome with normal nerve conduction studies. Muscle Nerve.

[4] Chen Y.-T., Williams L., Zak M.J., Fredericson M. (2016). Review of Ultrasonography in the Diagnosis of Carpal Tunnel Syndrome and a Proposed Scanning Protocol. J. Ultrasound Med..

[5] McDonagh C., Alexander M., Kane D. (2014). The role of ultrasound in the diagnosis and management of carpal tunnel syndrome: A new paradigm. Rheumatology.

[6] Al-Hashel J.Y., Rashad H.M., Nouh M.R., Amro H.A., Khuraibet A.J., Shamov T., Tzvetanov P., Rousseff R.T. (2015). Sonography in carpal tunnel syndrome with normal nerve conduction studies. Muscle Nerve.

[7] Aseem F., Williams J.W., Walker F.O., Cartwright M.S. (2017). Neuromuscular ultrasound in patients with carpal tunnel syndrome and normal nerve conduction studies. Muscle Nerve.

[8] Roghani R.S., Holisaz M.T., Norouzi A.A.S., Delbari A., Gohari F., Lokk J., Boon A.J. (2018). Sensitivity of high-resolution ultrasonography in clinically diagnosed carpal tunnel syndrome patients with hand pain and normal nerve conduction studies. J. Pain Res..

[9] Torres-Costoso A., Martínez-Vizcaíno V., Álvarez-Bueno C., Morales A.F., Cavero-Redondo I. (2018). Accuracy of Ultrasonography for the Diagnosis of Carpal Tunnel Syndrome: A Systematic Review and Meta-Analysis. Arch. Phys. Med. Rehabil..

[10] Cartwright M.S., Hobson-Webb L.D., Boon A.J., Alter K.E., Hunt C., Flores V.H., Werner R.A., Shook S.J., Thomas T.D., Do S.J.P. (2012). Evidence-based guideline: Neuromuscular ultrasound for the diagnosis of carpal tunnel syndrome. Muscle Nerve.

[11] Filius A., Scheltens M., Bosch H.G., van Doorn P.A., Stam H.J., Hovius S.E.R., Amadio P.C., Selles R.W. (2015). Multidimensional ultrasound imaging of the wrist: Changes of shape and displacement of the median nerve and tendons in carpal tunnel syndrome. J. Orthop. Res..

[12] Kuo T.-T., Lee M.-R., Liao Y.-Y., Chen J.-P., Hsu Y.-W., Yeh C.-K. (2016). Assessment of Median Nerve Mobility by Ultrasound Dynamic Imaging for Diagnosing Carpal Tunnel Syndrome. PLoS ONE.

[13] Wang Y., Filius A., Zhao C., Passe S.M., Thoreson A.R., An K.-N., Amadio P.C. (2014). Altered Median Nerve Deformation and Transverse Displacement during Wrist Movement in Patients with Carpal Tunnel Syndrome. Acad. Radiol..

[14] Park D. (2017). Ultrasonography of the Transverse Movement and Deformation of the Median Nerve and Its Relationships with Electrophysiological Severity in the Early Stages of Carpal Tunnel Syndrome. PM&R.

[15] Roomizadeh P., Eftekharsadat B., Abedini A., Ranjbar-Kiyakalayeh S., Yousefi N., Ebadi S., Babaei-Ghazani A. (2019). Ultrasonographic Assessment of Carpal Tunnel Syndrome Severity. Am. J. Phys. Med. Rehabil..

[16] Festen R.T., Schrier V.J., Amadio P.C. (2021). Automated Segmentation of the Median Nerve in the Carpal Tunnel using U-Net. Ultrasound Med. Biol..

[17] Chen X., Xie C., Chen Z., Li Q. (2019). Automatic Tracking of Muscle Cross-Sectional Area Using Convolutional Neural Networks with Ultrasound. J. Ultrasound Med..

[18] Loram I., Siddique A., Sanchez M.B., Harding P., Silverdale M., Kobylecki C., Cunningham R., Puccini M.B.S. (2020). Objective Analysis of Neck Muscle Boundaries for Cervical Dystonia Using Ultrasound Imaging and Deep Learning. IEEE J. Biomed. Health Inform..

[19] Hafiane A., Vieyres P., Delbos A. (2017). Deep learning with spatiotemporal consistency for nerve segmentation in ultrasound images. arXiv.

[20] Horng M.-H., Yang C.-W., Sun Y.-N., Yang T.-H. (2020). DeepNerve: A New Convolutional Neural Network for the Localization and Segmentation of the Median Nerve in Ultrasound Image Sequences. Ultrasound Med. Biol..

[21] Baby M., Jereesh A.S. Automatic nerve segmentation of ultrasound images. Proceedings of the 2017 International conference of Electronics, Communication and Aerospace Technology (ICECA).

[22] Huang C., Zhou Y., Tan W., Qiu Z., Zhou H., Song Y., Zhao Y., Gao S. (2019). Applying deep learning in recognizing the femoral nerve block region on ultrasound images. Ann. Transl. Med..

[23] Smistad E., Johansen K.F., Iversen D.H., Reinertsen I. (2018). Highlighting nerves and blood vessels for ultrasound-guided axillary nerve block procedures using neural networks. J. Med. Imaging.

[24] Zhao H., Sun N. Improved U-Net Model for Nerve Segmentation. Proceedings of the Image and Graphics.

[25] Abraham N., Illanko K., Khan N., Androutsos D. Deep Learning for Semantic Segmentation of Brachial Plexus Nervesin Ultrasound Images Using U-Net and M-Net. Proceedings of the 2019 3rd International Conference on Imaging, Signal Processing and Communication (ICISPC).

[26] Baka N., Leenstra S., Walsum T.V. (2017). Ultrasound Aided Vertebral Level Localization for Lumbar Surgery. IEEE Trans. Med. Imaging.

[27] Ronneberger O., Fischer P., Brox T. U-net: Convolutional networks for biomedical image segmentation. Proceedings of the International Conference on Medical Image Computing and Computer-Assisted Intervention.

[28] Chen L.C., Zhu Y., Papandreou G., Schroff F., Adam H. Encoder-decoder with atrous separable convolution for semantic image segmentation. Proceedings of the European Conference on Computer Vision (ECCV).

[29] Greenspan H., van Ginneken B., Summers R.M. (2016). Guest Editorial Deep Learning in Medical Imaging: Overview and Future Promise of an Exciting New Technique. IEEE Trans. Med. Imaging.

[30] Tajbakhsh N., Shin J.Y., Gurudu S.R., Hurst R.T., Kendall C.B., Gotway M.B., Liang J. (2016). Convolutional Neural Networks for Medical Image Analysis: Full Training or Fine Tuning?. IEEE Trans. Med. Imaging.

[31] Liu S., Wang Y., Yang X., Lei B., Liu L., Li S.X., Ni D., Wang T. (2019). Deep Learning in Medical Ultrasound Analysis: A Review. Engineering.

[32] He K., Gkioxari G., Dollár P., Girshick R. Mask r-cnn. Proceedings of the IEEE International Conference on Computer Vision.

[33] Kirillov A., Girshick R., He K., Dollár P. Panoptic feature pyramid networks. Proceedings of the IEEE Conference on Computer Vision and Pattern Recognition.

[34] Lin T.-Y., Dollár P., Girshick R., He K., Hariharan B., Belongie S. Feature pyramid networks for object detection. Proceedings of the IEEE Conference on Computer Vision and Pattern Recognition.

[35] Long J., Shelhamer E., Darrell T. Fully convolutional networks for semantic segmentation. Proceedings of the IEEE Conference on Computer Vision and Pattern Recognition.

[36] Mishra D., Chaudhury S., Sarkar M., Soin A.S. (2018). Ultrasound Image Segmentation: A Deeply Supervised Network with Attention to Boundaries. IEEE Trans. Biomed. Eng..

[37] Huang K., Zhang Y., Cheng H., Xing P., Zhang B. (2021). Semantic segmentation of breast ultrasound image with fuzzy deep learning network and breast anatomy constraints. Neurocomputing.

[38] Guo Y., Duan X., Wang C., Guo H. (2021). Segmentation and recognition of breast ultrasound images based on an expanded U-Net. PLoS ONE.

[39] Poudel P., Illanes A., Sheet D., Friebe M. (2018). Evaluation of Commonly Used Algorithms for Thyroid Ultrasound Images Segmentation and Improvement Using Machine Learning Approaches. J. Health Eng..

[40] Yu F., Koltun V. (2015). Multi-scale context aggregation by dilated convolutions. arXiv.

[41] Irfan R., Almazroi A., Rauf H., Damaševičius R., Nasr E., Abdelgawad A. (2021). Dilated Semantic Segmentation for Breast Ultrasonic Lesion Detection Using Parallel Feature Fusion. Diagnostics.

[42] Chen L.-C., Papandreou G., Schroff F., Adam H. (2017). Rethinking atrous convolution for semantic image segmentation. arXiv.

[43] Chen L.-C., Papandreou G., Kokkinos I., Murphy K., Yuille A.L. (2017). DeepLab: Semantic Image Segmentation with Deep Convolutional Nets, Atrous Convolution, and Fully Connected CRFs. IEEE Trans. Pattern Anal. Mach. Intell..

[44] Gómez-Flores W., Pereira W.C.D.A. (2020). A comparative study of pre-trained convolutional neural networks for semantic segmentation of breast tumors in ultrasound. Comput. Biol. Med..

[45] Cordts M., Omran M., Ramos S., Rehfeld T., Enzweiler M., Benenson R., Franke U., Roth S., Schiele B. The cityscapes dataset for semantic urban scene understanding. Proceedings of the IEEE Conference on Computer Vision and Pattern Recognition.

[46] Wu Y., Zhang R., Zhu L., Wang W., Wang S., Xie H., Cheng G., Wang F.L., He X., Zhang H. (2021). BGM-Net: Boundary-Guided Multiscale Network for Breast Lesion Segmentation in Ultrasound. Front. Mol. Biosci..

[47] Chiao J.-Y., Chen K.-Y., Liao K.Y.-K., Hsieh P.-H., Zhang G., Huang T.-C. (2019). Detection and classification the breast tumors using mask R-CNN on sonograms. Medicine.

[48] Atroshi I., Flondell M., Hofer M., Ranstam J. (2013). Methylprednisolone Injections for the Carpal Tunnel Syndrome. Ann. Intern. Med..

[49] Chang C.W., Wang Y.C., Chang K.F. (2008). A practical electrophysiological guide for non-surgical and surgical treatment of carpal tunnel syndrome. J. Hand. Surg. Eur. Vol..

[50] Wada K. (2016). Labelme: Image Polygonal Annotation with Python. https://github.com/wkentaro/labelme.

[51] Massa F., Girshick R. Maskrcnn-Benchmark: Fast, Modular Reference Implementation of Instance Segmentation and Object Detection Algorithms in PyTorch. https://github.com/facebookresearch/maskrcnn-benchmark.

[52] Xie S., Girshick R., Dollár P., Tu Z., He K. Aggregated residual transformations for deep neural networks. Proceedings of the IEEE Conference on Computer Vision and Pattern Recognition (CVPR).

[53] Ali F., El-Sappagh S., Islam S.R., Kwak D., Ali A., Imran M., Kwak K.-S. (2020). A smart healthcare monitoring system for heart disease prediction based on ensemble deep learning and feature fusion. Inf. Fusion.

[54] Wang X., Kong T., Shen C., Jiang Y., Li L. SOLO: Segmenting Objects by Locations. Proceedings of the Computer Vision—ECCV 2020.

[55] Srinivasu P., SivaSai J., Ijaz M., Bhoi A., Kim W., Kang J. (2021). Classification of Skin Disease Using Deep Learning Neural Networks with MobileNet V2 and LSTM. Sensors.

[56] Yang X., Yu L.Q., Wu L.G., Wang Y., Ni D., Qin J., Heng P.-A. Fine-grained recurrent neural networks for automatic prostate segmentation in ultrasound images. Proceedings of the Thirty-First AAAI Conference on Artificial Intelligence.

[57] Ijaz M.F., Attique M., Son Y. (2020). Data-Driven Cervical Cancer Prediction Model with Outlier Detection and Over-Sampling Methods. Sensors.

[58] Ijaz M.F., Alfian G., Syafrudin M., Rhee J. (2018). Hybrid Prediction Model for Type 2 Diabetes and Hypertension Using DBSCAN-Based Outlier Detection, Synthetic Minority Over Sampling Technique (SMOTE), and Random Forest. Appl. Sci..

